# Spatial Properties of Reactive Oxygen Species Govern Pathogen-Specific Immune System Responses

**DOI:** 10.1089/ars.2020.8027

**Published:** 2020-03-24

**Authors:** Eunice E. To, John J. O'Leary, Luke A.J. O'Neill, Ross Vlahos, Steven Bozinovski, Christopher J.H. Porter, Robert D. Brooks, Doug A. Brooks, Stavros Selemidis

**Affiliations:** ^1^ Program in Chronic Infectious and Inflammatory Diseases, Oxidant and Inflammation Biology Group, School of Health and Biomedical Sciences, College of Science, Engineering & Health, RMIT University, Melbourne, Australia.; ^2^ Infection and Immunity Program, Department of Pharmacology, Biomedicine Discovery Institute, Monash University, Clayton, Australia.; ^3^ Discipline of Histopathology, School of Medicine, Trinity Translational Medicine Institute (TTMI), Trinity College Dublin, Dublin, Ireland.; ^4^ Sir Patrick Dun's Laboratory, Central Pathology Laboratory, St James's Hospital, Dublin, Ireland.; ^5^ Emer Casey Research Laboratory, Molecular Pathology Laboratory, The Coombe Women and Infants University Hospital, Dublin, Ireland.; ^6^ CERVIVA Research Consortium, Trinity College Dublin, Dublin, Ireland.; ^7^ School of Biochemistry and Immunology, Trinity Biomedical Sciences Institute, Trinity College Dublin, Dublin, Ireland.; ^8^ ARC Centre of Excellence in Convergent Bio-Nano Science and Technology, Monash Institute of Pharmaceutical Sciences, Monash University, Parkville, Australia.; ^9^ Drug Delivery Disposition and Dynamics, Monash Institute of Pharmaceutical Sciences, Monash University, Parkville, Australia.; ^10^ School of Pharmacy and Medical Sciences, University of South Australia Cancer Research Institute, University of South Australia, Adelaide, Australia.

**Keywords:** reactive oxygen species, NADPH, endosome, mitochondria

## Abstract

***Significance:*** Reactive oxygen species (ROS) are often considered to be undesirable toxic molecules that are generated under conditions of cellular stress, which can cause damage to critical macromolecules such as DNA. However, ROS can also contribute to the pathogenesis of cancer and many other chronic inflammatory disease conditions, including atherosclerosis, metabolic disease, chronic obstructive pulmonary disease, neurodegenerative disease, and autoimmune disease.

***Recent Advances:*** The field of ROS biology is expanding, with an emerging paradigm that these reactive species are not generated haphazardly, but *instead* produced in localized regions or in specific subcellular compartments, and this has important consequences for immune system function. Currently, there is evidence for ROS generation in extracellular spaces, in endosomal compartments, and within mitochondria. Intriguingly, the specific location of ROS production appears to be influenced by the type of invading pathogen (*i.e.*, bacteria, virus, or fungus), the size of the invading pathogen, as well as the expression/subcellular action of pattern recognition receptors and their downstream signaling networks, which sense the presence of these invading pathogens.

***Critical Issues:*** ROS are deliberately generated by the immune system, using specific NADPH oxidases that are critically important for pathogen clearance. Professional phagocytic cells can sense a foreign bacterium, initiate phagocytosis, and then within the confines of the phagosome, deliver bursts of ROS to these pathogens. The importance of confining ROS to this specific location is the impetus for this perspective.

***Future Directions:*** There are specific knowledge gaps on the fate of the ROS generated by NADPH oxidases/mitochondria, how these ROS are confined to specific locations, as well as the identity of ROS-sensitive targets and how they regulate cellular signaling.

## Why Is There a Need for Reactive Oxygen Species Compartmentalization?

As the term reactive oxygen species (ROS) implies, ROS are highly reactive molecules and to a large degree their site of generation will therefore determine their site of action; but nonetheless, this must avoid off target effects. For instance, superoxide anion, often referred to as the “parent” ROS, is generated by a variety of enzymatic and nonenzymatic systems in either a deliberate manner by enzymes whose primary function is to generate ROS (*i.e.*, NADPH oxidases) or inadvertently as a consequence of a dysfunctional variant of the system, such as uncoupled nitric oxide synthase or in mitochondria. However, irrespective of how the superoxide is generated, it is rapidly converted to hydrogen peroxide (H_2_O_2_), either spontaneously from the reaction of two superoxide molecules or *via* enzymatic catalysis using the superoxide dismutase (SOD) family of enzymes; in a rapid process described by a rate constant of ∼10^8^ *M*/s. Moreover, superoxide can react with another important molecule, nitric oxide (NO), to give rise to peroxynitrite (ONOO^−^), a very powerful oxidant with pleiotropic actions (42). The reaction between superoxide and NO is the fastest known biological reaction at ∼5 × 10^9^ *M*/s and therefore is highly diffusion limited. Thus, the overall level of any particular ROS in a biological system is strictly controlled by tight regulation of its enzymatic production, as well as its removal or metabolism by highly efficient antioxidant enzymes such as SOD, catalase, glutathione peroxidase and peroxiredoxins, or soluble antioxidants such as glutathione and ascorbate. This ROS chemistry has been the subject of many comprehensive reviews by experts in the field (50, 51) and therefore will not be covered in this perspective. However, it is becoming increasingly clear that cells have evolved ways to compartmentalize ROS production, which ensures localized signaling and restricts inadvertent effects of ROS on other critical cellular machinery. For example, there is emerging evidence that ROS production occurs in specific compartments of the cell in response to invading microorganisms. In the following sections, we will describe several scenarios of distinct sites for ROS generation, with a brief description of how these processes occur and particularly the impact that they have on innate immune system function.

## Phagosomal NOX2 Oxidase-Derived ROS Response to Small Bacteria and Fungi

**FIG. 1.**
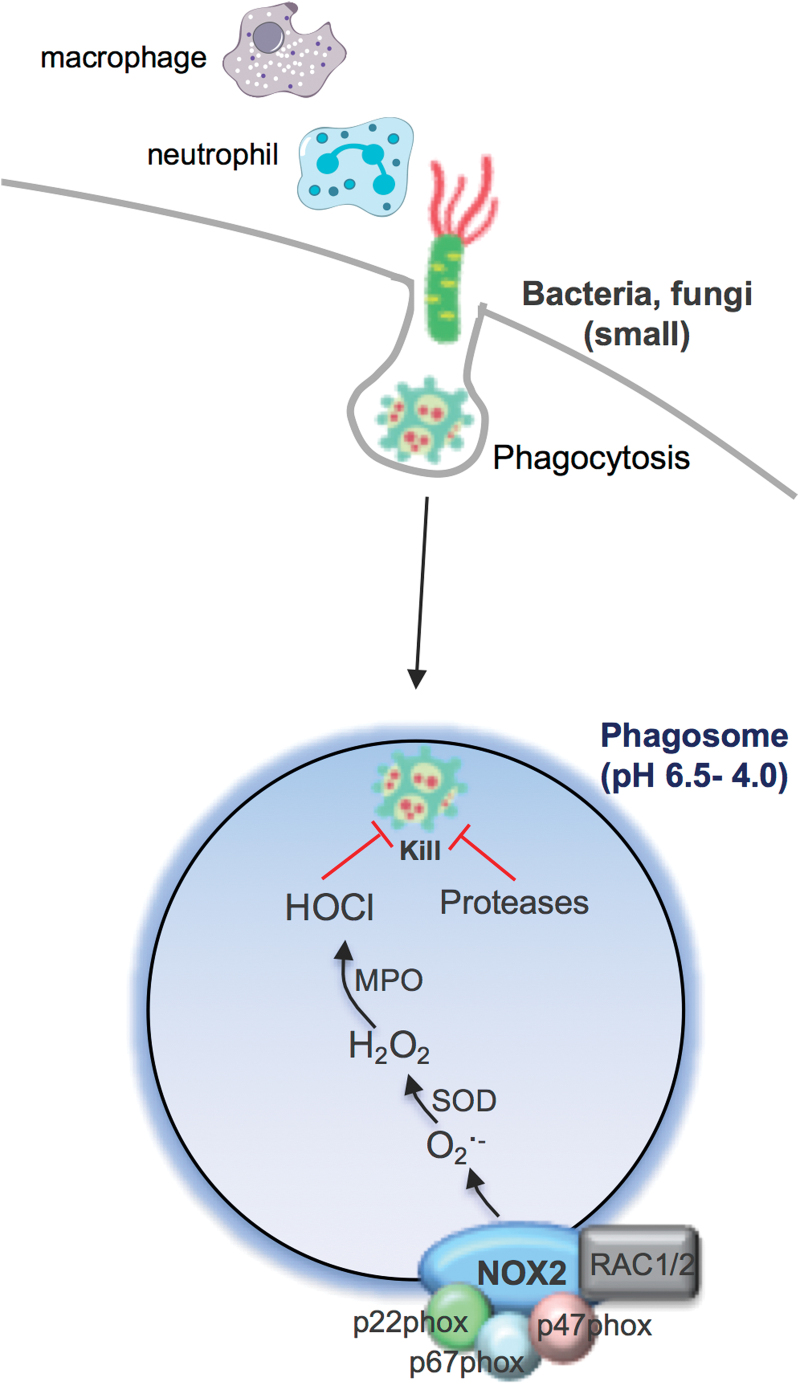
**Schematic representation of the phagosomal ROS production to “small” bacteria and fungi.** Bacteria and fungi can be internalized *via* phagocytosis initiating phagosomal superoxide production by NOX2 oxidase and then conversion to H_2_O_2_ and HOCl *via* MPO (in neutrophils, mainly). Along with proteases, ROS are thought to “kill” the invading bacteria or fungi. H_2_O_2_, hydrogen peroxide; HOCl, hypochlorous acid; MPO, myeloperoxidase; ROS, reactive oxygen species. Color images are available online.

Unquestionably, the most highly characterized subcellular compartment relating to ROS production is the phagosome. In this specialized endocytic compartment, high concentrations of ROS can be achieved and this has been referred to as an “oxidative burst.” This process occurs primarily in neutrophils and macrophages after engulfing bacteria or fungi and provides an initial line of host defense against these invading pathogens (Fig. 1). This ROS generation is the primary function of an enzyme family called NADPH oxidases and is distinct from other enzymes that produce ROS either as a by-product of their normal catalytic activity or due to abnormal function during disease (3, 5, 7, 10, 11, 22, 25, 33, 39). NADPH oxidase enzymes are expressed in most mammalian cell types, where they catalyze the reduction of molecular oxygen to generate superoxide (and/or H_2_O_2_) in various intracellular and extracellular compartments. NADPH oxidases are membrane-bound enzymes that utilize electrons from cytosolic NADPH to catalyze ROS production, and there are seven NADPH oxidase isoforms (5, 10). These NADPH oxidases are expressed in different subcellular membrane compartments, including the phagosome, endosome, mitochondria, endoplasmic reticulum (ER), and nucleus as well as in association with the cell surface, for ROS production in the extracellular space (10). However, a functional NADPH oxidase is not always present in each of these compartments, and this is part of the regulatory system that ensures that ROS are only produced under specific conditions. For example, when neutrophils or macrophages phagocytose bacteria or fungi, this initiates a complex series of events, which includes the assembly of the prototypical NOX2-containing NADPH oxidase enzyme complex at the phagosomal membrane (Fig. 1). This results in the capacity for electron transfer from NADPH to the NOX2 catalytic subunit, which comprises a single flavin adenine dinucleotide (FAD) molecule bound to the extended carboxy-terminal tail of NOX2, and two prosthetic heme groups attached to histidine residues within the transmembrane region. Oxygen bound to heme acts as the final electron acceptor resulting in the release of superoxide from the enzyme within the phagosome compartment. This is the natural direction of superoxide generation by phagosomal NOX2 NADPH oxidase, owing to the topology of the NOX2 catalytic subunit and the conduit of electron accepting moieties found within this subunit.

Even though it has been more than 80 years since the initial discovery of the phagocytic oxidative burst by Baldridge and Gerard, it is still largely unknown how the generation of superoxide/ROS within the phagosome results in an antibacterial response and how it impacts on the immune system (4). This topic has been hotly debated, and multiple scenarios have been emphasized. The first and more widely accepted view is that superoxide generated within the phagosome is converted by a series of enzymes to H_2_O_2_, that is, SOD and then to the more powerful oxidizing species hypochlorous acid (and other derivatives) by myeloperoxidase (MPO). This powerful oxidizing environment effectively “bleaches” the bacterium or fungus and thereby eliminates the pathogen (33). However, this widely held view of ROS being the ultimate mediator for pathogen killing has been contested, particularly by Segal (37). Indeed, it was demonstrated that the NOX2-containing oxidase of the neutrophil is critical for pumping electrons into the phagocytic vacuole, and in the process, inducing a charge differential across the membrane that must be compensated. The compensating ion movement produces conditions within the vacuole that are favorable to bacterial killing and digestion by hydrolase enzymes that are released into the vacuole from cytoplasmic granules, and not killing by ROS directly. Perhaps, the most compelling data arguing against ROS and oxidized halides serving as direct microbicidal agents are provided by mice with suppressed neutral granule proteases, cathepsin G, and elastase (34). While these mice display normal superoxide/ROS generation and normal iodination, as a consequence of normal MPO activity, they are unable to kill *Staphylococcus aureus* and *Candida albicans*, with a killing defect at least as severe as that in the chronic granulomatous disease (CGD) mouse lacking p47^*phox*^.

## Extracellular NOX2 Oxidase-Derived ROS for Large Bacteria and Fungi

**FIG. 2.**
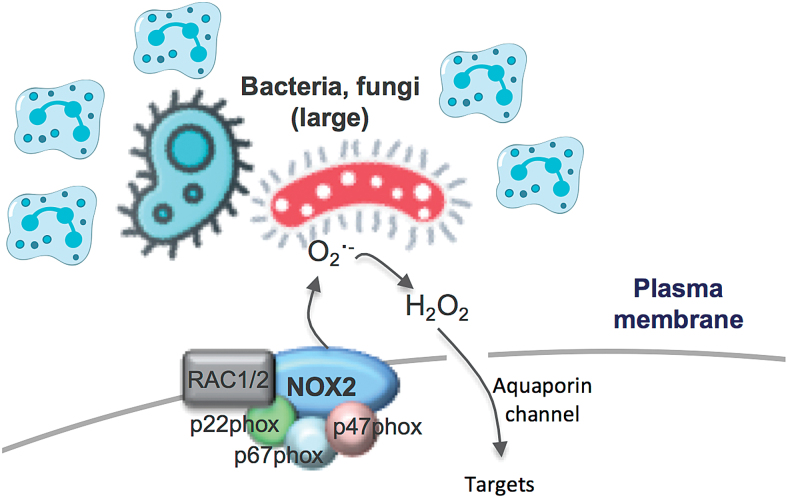
**Schematic representation of extracellular ROS production *via* NOX2 oxidase.** Large bacteria and fungi cause frustration of neutrophils resulting in extracellular ROS production. Also shown is extracellular H_2_O_2_ permeating plasma membrane *via* aquaporin channels. Color images are available online.

The activation of NOX2 oxidase occurs within seconds of degranulation and after vacuole closure, but in this case, the superoxide is not detected in the extracellular space of the cell that is phagocytosing the bacterium. However, if neutrophils are “frustrated” and the process of phagocytosis is delayed or engulfment is not possible, superoxide can be detected extracellularly (Fig. 2). It has been proposed that the size of bacteria and fungi influences whether ROS production occurs within the phagosome or extracellularly (48), and this presumably relates to the capacity to easily phagocytose the target pathogen. The localization of ROS generated in response to pathogens of differing size is proposed to influence immune signaling, interleukin (IL)-1β expression, and the redox state of nuclear factor kappa B (NF-κB). For instance, small bacteria and fungi that are phagocytosed activate NOX2 to produce ROS intracellularly, presumably within the phagosome (48). In these studies, the p50 subunit of NF-κB underwent oxidation, and the inflammasome complex coupled with IL-1β expression was suppressed, in a manner that was blocked by diphenyleneiodonium chloride (DPI), a widely used NADPH oxidase inhibitor. While inhibition by DPI implies NADPH oxidase as a source of ROS, this interpretation needs to be made cautiously, because at the concentration used, that is, 10 μ*M*, DPI will inhibit other FAD-containing enzymes such as endothelial nitric oxide synthase, xanthine oxidase, and proteins of the mitochondrial electron transport chain (ETC) (1). In contrast to small microbes, neutrophils that fail to engulf larger microbes, such as *C. albicans*, activate plasma membrane-bound NOX2 oxidase and produce ROS in the extracellular space. Extracellular ROS has been suggested to be delivered to this site by endosomes undergoing exocytosis at the cell surface (*i.e.*, endosomes are contiguous with the cell surface and contain NOX2). However, this NOX2 oxidase is assembled at the plasma membrane, and while the production of extracellular ROS is believed to be involved in killing the *Candida*, it also prevents oxidation of p50, which amplifies IL-1β expression. Elevated IL-1β can stimulate stronger recruitment of neutrophils, which can then form clusters around large microbes.

Although these findings highlight the potential importance of ROS localization in shaping the immune response, some of the points raise additional questions. In particular, how phagosomal ROS, generated in response to engulfed bacteria and fungi, find its way from the phagosome to the cytosol to cause oxidation of p50 and how this suppresses the inflammasome complex. The ROS detection techniques employed by Warnatsch *et al.* (48) fail to provide definitive evidence for phagosome ROS production or subsequent phagosome membrane permeation of any particular ROS. In fact, luminol and isoluminol were used to measure total ROS and extracellular ROS, respectively. Luminol is less polar and less hydrophilic than isoluminol, is capable of crossing biological membranes, and is suitable for detecting both intracellular and extracellular ROS. By contrast, isoluminol only slightly permeates cell membranes, due to its polar and hydrophilic nature, although its internalization into phagosomes or endosomes cannot be ruled out. Therefore, one cannot make the definitive conclusion that isoluminol is predominantly measuring ROS in the extracellular space. Even the suppression of the isoluminol chemiluminescence by the “so-called” extracellular scavengers such as SOD and catalase does not provide definitive evidence for extracellular ROS production because both SOD and catalase are readily internalized by endocytosis. Despite ongoing uncertainties in this field, the study by Warnatsch *et al.* (48) highlights some exciting developments in the field of redox compartmentalized signaling and an evident gap in the knowledge base for this fundamental biological process.

The type of ROS influencing intracellular targets within the cytosol is also undefined. Superoxide, which is anionic, will most likely be confined within the phagosome, although it may permeate the phagosomal membrane *via* specialized anionic Cl^−^ channels (32). Perhaps, a better candidate is the direct derivative of superoxide, H_2_O_2_, which is not charged and has the capacity to permeate cell membranes to some degree. Despite strong evidence that ROS have the capacity to permeate cell membranes, it still begs the question, why would a cell utilize such an intricate system to impact on cell signaling? It seems counterintuitive that a complex enzyme system, such as NOX2 oxidase, has functionality in the phagosomal membrane only for the ROS it generates within the phagosome to ultimately have targets *outside* this compartment? Similarly, it is difficult to imagine why cells would deliberately generate ROS extracellularly, only for that ROS to then be shuttled back in, *via* aquaporin channels, to find targets within the cell? Moreover, this might be further hampered by the half-life of any extracellular ROS, which will likely be strongly influenced by the extracellular milieu. For instance, superoxide released into the extracellular space could diffuse rapidly from its site of generation and, at the same time, be exposed to high levels of extracellular antioxidants such as extracellular SOD. If any H_2_O_2_ is generated by dismutation of superoxide and permeates (or enters by aquaporin channels) the cell membrane, it will potentially interact with catalase and glutathione peroxidase in the cytosol. These factors make it unlikely that ROS generated extracellularly will function as intracellular signaling molecules, although the region of membrane at the cell surface could be internalized into endosomes to facilitate downstream signaling.

## Endosomal ROS Production: Virus Driven

Endosomes can be generated by cell surface invagination and are involved in a wide range of cellular functions, including extracellular uptake, enzymatic degradation, intracellular transport, receptor recycling, neurotransmission, secretion, and intracellular signaling. Endosomes have an integral role in immune function through, for example, pathogen phagocytosis and phagosome maturation/degradation, toll-like receptor (TLR) function, antigen processing, the secretion of antimicrobial peptides and cytokines, antibody delivery, early endosome intracellular signaling, and late endosome control of immune signaling. Given the specific role of endosomes in innate immune function, it is not surprising that specialist killing mechanisms have been developed by these functional compartments to help deal with internalized pathogens.

As eluded to above, historically, there has been a wide interest into how phagosomal or extracellular NOX2 oxidase-derived ROS promote the killing of bacteria and fungi. However, for unknown reasons, less attention has been paid to virus infection, and as a consequence, several important key knowledge gaps were recently identified. First, what is the subcellular compartment for ROS generation in response to virus infection and what is the relevant enzymatic source of ROS? Second, what are the molecular targets of ROS and are these within the specific organelle in which the ROS are generated? Third, does ROS generation impact on antiviral signaling and clearance? Finally, is it possible to target this ROS response to virus infection with organelle-specific pharmacological inhibitors? Intriguingly, several recent seminal studies have shown that NOX2 oxidase-derived ROS *do not* eliminate viruses in a manner analogous to that for bacteria and fungi. In contrast, in the absence of NOX2, viruses such as influenza A cause substantially less lung injury and dysfunction, and this leads to substantially lower viral burden (16, 23, 25, 41, 43, 46, 47). Therefore, NOX2 oxidase-derived ROS *promote* rather than inhibit viral infection. We recently provided evidence for how viruses cause ROS production and how these highly reactive oxygen molecules actually exacerbate viral disease (43).

**FIG. 3.**
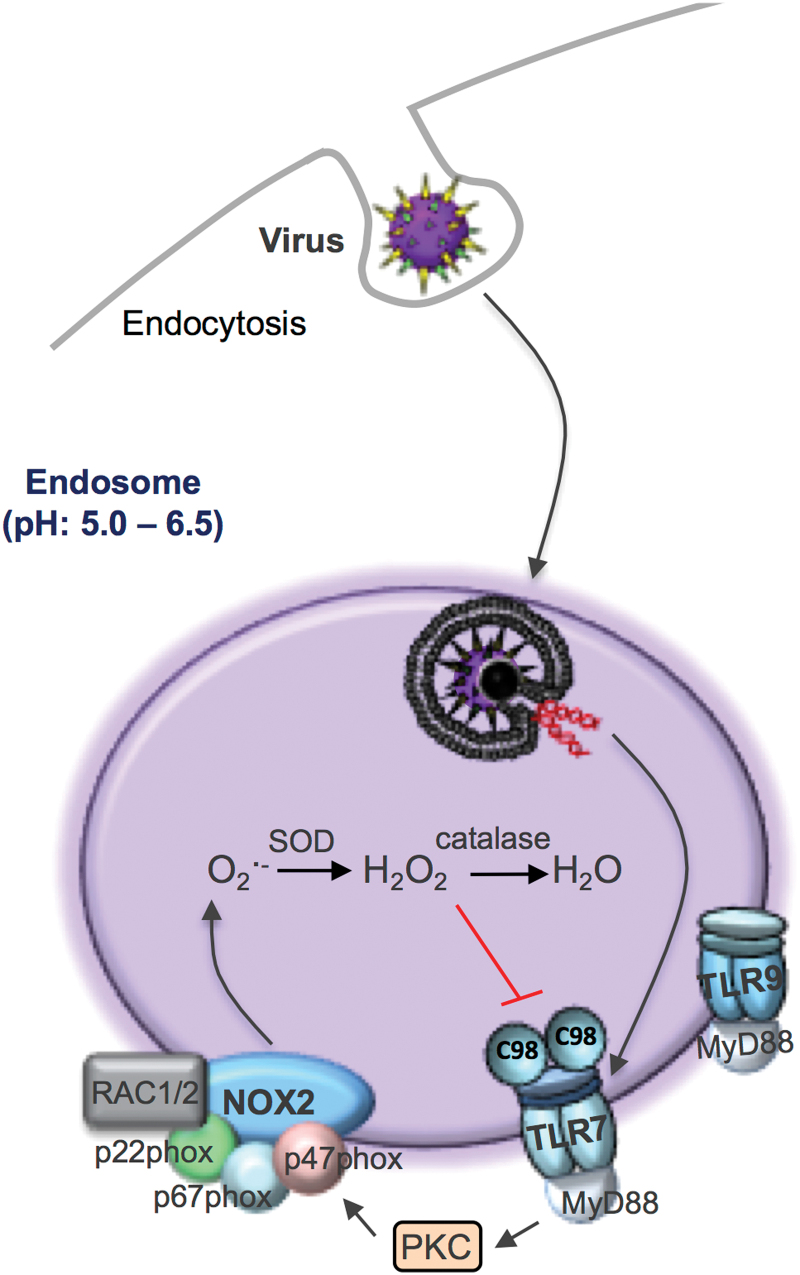
**Schematic representation of the endosomal ROS production pathway to virus infection.** Viruses that internalize *via* endocytosis are sensed by endosomally located TLRs, in particular TLR7 for ssRNA viruses and TLR9 for DNA viruses (for clarity, endosome TLR3 is not shown). Activation of TLR7-MyD88 mediates ROS generation *via* PKC, which triggers the phosphorylation of specific serine residues on the p47^phox^ subunit that promotes the assembly of a functional NOX2 oxidase enzyme. The H_2_O_2_ generated within endosomal compartments by NOX2 targets cysteine 98 (C98) on the ectodomain of TLR7. PKC, protein kinase C; TLR, toll-like receptor. Color images are available online.

It is well understood that after binding to the plasma membrane, many viruses enter cells by endocytosis. Within the endosome, viral RNA (or DNA depending on the virus) is detected by endosomal pattern recognition receptors, including TLR3, TLR7, and TLR9 (Fig. 3). The specific receptor interaction depends on either the Group (I–V) or genomic orientation (*i.e.*, ssRNA, dsRNA, or DNA) of the virus, and this triggers an immune response characterized by type I interferon (IFN) and IL-1β production. We have provided evidence that NOX2 oxidase is expressed in endosomes (43), which is consistent with some observations showing co-localization of NOX2 and NOX1 with EEA1- and RAB-5-positive endosomes (21, 24, 29, 32). Importantly, we showed that viruses which are internalized by endocytosis, including ssRNA viruses *irrespective* of their classification, such as influenza A virus, respiratory syncytial virus, rhinovirus, Dengue virus and HIV, as well as the DNA viruses, vaccinia virus, and herpes simplex virus cause activation of NOX2 oxidase and the production of ROS in endosomes. This response was time-dependent and varied according to the dose of the virus. Activation of endosomal NOX2 by ssRNA viruses and DNA viruses was suppressed by the absence of TLR7 and TLR9, respectively, and were the result of protein kinase C (PKC) activation. Importantly, we showed that influenza exposure to wild type (WT) macrophages elevated cytosolic PKC activity within 5 min, and this response was absent in TLR7^−/−^ macrophages and in WT macrophages treated with the inhibitors dynasore or bafilomycin A. The spatial and temporal aspects of endosome ROS generation in response to virus infection accurately reflect viral internalization by endocytosis, endosome compartmentalization, and pattern recognition receptor signaling network activation. These studies have defined for the first time that endosomes are specialized compartments for ROS production and redox signaling in response to virus infection.

We also showed that endosomal NOX2 oxidase activity resulted in the suppression of expression of key antiviral cytokines (43). This was most likely attributed to H_2_O_2_, as inactivation of endosomal H_2_O_2_ by exogenous catalase administration markedly elevated type I IFN and IL-1β expression. The effect of catalase was impaired by the endocytosis inhibitor Dynasore and by the absence of both TLR7 and the chaperone UNCB93. We questioned whether H_2_O_2_ that was released by endosomal NOX2 oxidase targets cysteine residues on protein domains of TLR7 and whether this regulates receptor activity and causes the exposure of critical residues upon activation within endosomal compartments. Consistent with this hypothesis, we showed that H_2_O_2_ produced by endosomal NOX2 oxidase is likely to modify a single and evolutionarily conserved unique cysteine residue, that is, Cys98 located on the endosomal face of TLR7. Notably, this can result in dampening of the antiviral cytokine response and the humoral immune response. Cys98 forms a disulphide bond with Cys475 on TLR7, which is critical for receptor activation (52). Potentially, this signifies Cys98 of TLR7 as a novel redox sensor that may dampen immune function during viral infections. Subsequently, we demonstrated that the suppression of ROS production during viral infection restored immune function and enhanced viral clearance (43). The confinement of the ROS source (NOX2 oxidase) and ROS target (C98-TLR7) within the endosome is an important example of redox signaling and is fundamentally consistent with the most likely way that highly reactive ROS are used to influence cell signaling processes.

Overall, the current status of the field of endosome ROS biology is still in its infancy, and further investigation will be required to establish critical biochemical and functional implications of endosomal ROS production in the context of not only viral infections but also receptor function in general. This could have even broader relevance for the function of, and signaling from, plasma membrane-bound receptors such as G protein-coupled receptors and growth factor receptors, which are also internalized by endocytosis.

## Mitochondrial ROS

**FIG. 4.**
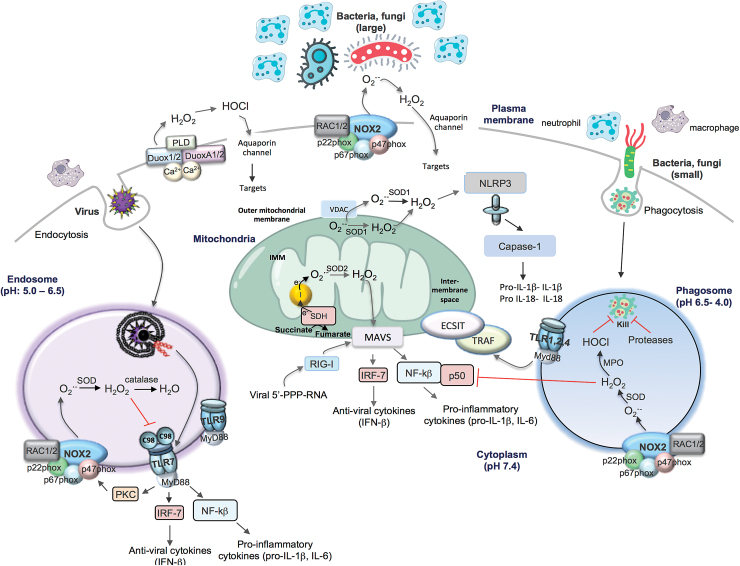
**Schematic representation of the complexities of the various subcellular compartments of ROS production and ROS targets that impact on immune system function.** Viruses that internalize *via* endocytosis are sensed by endosomally located TLR7 for ssRNA viruses and TLR9 for DNA viruses. In addition to driving endosomal NOX2 oxidase, activation of TLR7 drives transcription factors including IRF-7 and NF-κB that are essential for the induction of antiviral cytokines (IFNα/β) and proinflammatory cytokines (IL-1β, IL-6), respectively. TLR7 mediates ROS generation *via* PKC, and the H_2_O_2_ generated within endosomal compartments by NOX2 targets the TLR7 cysteine 98 on the ectodomain to dampen antiviral signaling networks. Large bacteria and fungi can stimulate the production of extracellular superoxide at the level of the plasma membrane. Alternatively, bacteria and fungi (small) can be internalized *via* phagocytosis initiating phagosomal superoxide production by NOX2 oxidase and then conversion to H_2_O_2_ and HOCl *via* MPO (in neutrophils, mainly). Along with proteases, ROS are thought to “kill” the invading bacteria or fungi. In addition, phagosomal ROS oxidize the p50 protein subunit of NF-κB leading to ubiquitination and degradation of p50. Within the phagosome, engagement of bacterial sensors TLR1, TLR2, or TLR4 causes translocation of TRAF to the mitochondria that binds to ECSIT, facilitating the generation of mtROS to enhance bacterial killing. Mitochondrial H_2_O_2_ targets MAVS for activation of IRF-7 and NF-κB. mtROS may also activate NLRP3 leading to inflammation activation. ECSIT, evolutionarily conserved signaling intermediate in toll pathways; IFN, interferon; IL, interleukin; IRF-7, interferon regulatory factor 7; MAVS, mitochondrial antiviral signaling; mtROS, mitochondrial ROS; NF-κB, nuclear factor kappa B; NLRP3, NACHT, LRR, and PYD domains-containing protein 3; TRAF, tumor necrosis factor receptor-associated factor. Color images are available online.

Although mitochondria are organelles for energy metabolism and accommodate the tricarboxylic acid cycle and oxidative phosphorylation steps necessary for efficient ATP generation, it has recently been demonstrated that mitochondria are also critical hubs for the regulation of the immune response following pathogen exposure that involves the generation of ROS (Fig. 4) (30, 31). Superoxide is generated on both sides of the inner mitochondrial membrane and hence arises in the matrix or the intermembrane space. The electron flow through the mitochondrial ETC to complex IV results in their final deposition into molecular oxygen to form water. However, electrons can also react with oxygen at sites in the ETC to form superoxide/H_2_O_2_. Complexes I and III are often regarded as the major sites of mitochondrial ROS (mtROS) production, but up to 10, other mitochondrial enzymes also contribute, including complex II. As seen in other compartments, superoxide is converted to H_2_O_2_ by SOD enzymes, in this instance SOD1, which is expressed in the intermembrane space, and SOD2, which is expressed in the matrix. The functional effects of mtROS are varied, but there has been a great deal of interest in specific effects on innate immunity.

Mitochondria have recently been touted as critical platforms for innate immune activation during bacterial and viral infection. For instance, lipopolysaccharide (LPS) exposure changes the overall phenotype of macrophages toward a proinflammatory state, and this is most likely due to alterations in the processes of oxidative phosphorylation occurring in the mitochondria. LPS causes a switch from oxidative phosphorylation to glycolysis, and this “collapse” of the mitochondria results in an increase in mitochondrial membrane potential, and the “slowing” or reversal of electron transport (aka RET) through the ETC at complex I. This results in significant elevations of superoxide generation at complex I. Succinate is critical in this process, and its accumulation and oxidation by succinate dehydrogenase drive mtROS via RET (30).

Several functional consequences of mitochondria ROS generation have been demonstrated including activation of the mitochondrial antiviral signaling (MAVS) protein, which is a key signaling protein activated by the viral RNA sensors retinoic acid inducible gene I (RIG-I) and melanoma differentiation-associated protein 5 (MDA-5). The locality of MAVS close to the mitochondrial membrane would be conducive for ROS activation that occurs within its vicinity. This process activates critical antiviral signaling systems that help clear viral infections. mtROS can also drive MAVS oligomerization, leading to the production of type I interferon, independent of RNA sensing, which suggests that MAVS might be a key sensor of mtROS that act to promote host defense and inflammation in general (8). mtROS can also activate the inflammasome, although this has been an area of great controversy. This has been largely attributed to a lack of specific tools to measure ROS and to manipulate ROS production from NADPH oxidases and mitochondria. Some of the early studies suggest that ROS promote inflammasome activation based on evidence of sensitivity to high concentrations of DPI (*i.e.*, 20 μ*M*). The sensitivity of DPI was suggestive of NADPH oxidase, although as mentioned above this concentration of DPI can be problematic (9). Indeed, a series of follow-up studies showed that monocytes from CGD patients who have defective NOX2 and p22phox (defect in NOX1–NOX4) had similar inflammasome activity or in some cases elevated activity (28, 44). These findings suggest that NOX-derived ROS are either redundant or suppress inflammasome activity. Also, consistent with ROS suppressing the inflammasome, there is evidence that SOD1-deficient macrophages, which fail to inactivate superoxide, secrete lower levels of active IL-1β upon inflammasome stimulation. A potential reason for this is a reversible oxidation of cysteines 362 and 397 on caspase-1, suppressing its activity (27). More recent studies now claim that ROS promote inflammasome activity and the consensus that appears to be forming is that the ROS are mitochondrial in origin (49, 53). Thus, inhibition of complex I or III of the mitochondrial respiratory chain, which is known to result in ROS generation, increased NLRP3 inflammasome activation, and this coincided with NLRP3 and ASC co-localization with mitochondria. NLRP3 of the inflammasome complex drives IL-1β expression, which is an important proinflammatory cytokine (53). Overall, the accumulation of succinate within the mitochondria due to LPS (and bacterial infection) and TLR1, TLR2, and TLR4 activation resulting in mtROS production represents an important process that reprograms macrophages, such that they are no longer ATP generators but mtROS generators, which would serve them well for promoting an acute inflammation in response to infection.

mtROS have also been suggested to regulate phagosomal ROS levels for antibacterial purposes, although the mechanisms are still undefined. The bacterial sensors TLR1, TLR2, and TLR4, but not the viral endosome located TLR3, TLR7, or TLR9, have been shown to stimulate mtROS by triggering the translocation of the TLR signaling adaptor tumor necrosis factor receptor-associated factor 6 (TRAF6) to the mitochondria, where it associates with the protein ECSIT (evolutionarily conserved signaling intermediate in toll pathways) and is implicated in mitochondrial respiratory chain assembly (49). The mtROS generated is proposed to be involved in bacterial killing, by contributing to phagosomal ROS levels in collaboration with NOX2 oxidase. This still remains a controversial idea and there is insufficient evidence to definitively support how this would occur. First, there is still an uncertainty of what is the driving (electrical, chemical) force for mtROS to traverse the inner and outer mitochondrial membranes, and then, these membranes to enter the phagosome where there is such a powerful ROS-generating system, the NOX2 oxidase, already in place. Second, what is the identity of the ROS that traverse across these membranes? It has been suggested to be H_2_O_2_, but its fate is governed by antioxidant enzymes expressed within the mitochondrial space, cytosol, and other organelles such as peroxisomes, and this has therefore yet to be directly established. Third, what are the temporal aspects of mtROS production and do these coincide with the kinetics of microbial killing due to NOX2 oxidase? In neutrophils, a heavily opsonized particle is taken into the phagocytic vacuole within 20 s and killing is almost immediate with NOX2 oxidase activation occurring within 20–45 s of vacuole closure. mtROS production is therefore unlikely to occur before phagosomal NOX2 oxidase activation. Indeed, the two key studies implicating TLR1, TLR2, and TLR4 activation leading to mtROS production only showed a significant elevation in mtROS after 3, 6, and 16 h of TLR activation (13, 49). Moreover, the kinases Mst1 and Mst2 function to control ROS production by regulating mitochondrial trafficking and mitochondrion–phagosome juxtaposition (13). Mst1 and Mst2 activate Rac GTPase to promote TLR-triggered assembly of the TRAF6-ECSIT complex that is required for mitochondrial recruitment to phagosomes (13). However, even the temporal aspects of these processes of mitochondrion–phagosome juxtaposition and Mst1- and Mst2-dependent Rac GTPase activation are not clear. Fourth, the phagosomal NOX2 oxidase can generate from ∼1 to 4 *M* in the vacuole with the steady-state concentration estimated to be in the micromolar range. Although, we do not know for sure, it is unlikely that mitochondria will generate levels of ROS that will significantly influence the overall level of ROS in the phagosome.

## Immune Responses by Nonphagocytic NADPH Oxidase Isoforms

The discussion thus far has been centered largely on NOX2 oxidase expressed by immune cells, the reason being that NOX2 oxidase is the prototypical oxidase that has traditionally been considered *the* primary mediator of ROS production by the immune system in response to external pathogens. However, there is accumulating evidence that the other NADPH oxidase enzyme family members, that is, DUOX, NOX4, and NOX1, are expressed in epithelium (airway and gut) and provide a tissue barrier to danger signals such as infection with certain bacteria and viruses (12, 38, 45). For example, Dual oxidase 2 (DUOX2) is the most abundant isoform present in the gastrointestinal epithelium, is expressed at much higher levels than DUOX1, and generates extracellular ROS to protect against *Helicobacter pylori* colonization (14). It is thought that DUOX2 is engaged by TLRs and some intracellular NODs (*i.e.*, NOD2) to provide extracellular H_2_O_2_ to lactoperoxidase to produce antimicrobial hypothiocyanite ions. Moreover, both DUOX1 and DUOX2 are abundant in the respiratory epithelium and generate high levels of extracellular H_2_O_2_ to exert antibacterial and antiviral responses, although the mechanisms are not well characterized (17, 20, 45). Thus far, the evidence implicates DUOX2 as the predominant functionally active antibacterial and antiviral DUOX in the airways. For example, influenza A virus infection has been shown to lead to a coordinated DUOX2 upregulation and DUOX-mediated ROS generation. Interference with H_2_O_2_ production and ROS signaling by oxidase inhibition or H_2_O_2_ decomposition augmented influenza A virus (IAV) replication. A nuclear pool of DUOX enzymes participated in the regulation of the spliceosome, which promotes alternative splicing of viral transcripts and controls the assembly of viable virions. *In vivo* silencing of DUOX increased the viral load on intranasal infection with 2009 pandemic H1N1 influenza virus (40). In separate studies, overexpression of DUOX2 suppressed influenza A virus replication in the nasal epithelium, and this was associated with elevations in the viral sensors RIG-I, MDA-5, and type I IFNs, suggesting an important antiviral and protective role for DUOX2-derived ROS against IAV-induced morbidity (16, 18, 20). However, the issues raised above of how extracellular ROS influence intracellular signaling systems apply equally strongly to ROS generated by DUOX enzymes. Indeed, DUOX-derived H_2_O_2_ causes reversible cysteine oxidation of critical proteins such as nonreceptor TK Src and epidermal growth factor receptor (45). Importantly, these mechanisms are largely unaffected by extracellular catalase (45), suggesting that DUOX-dependent signaling originates primarily from activation of intracellularly localized DUOX1 such as at ER membranes or perhaps from internalized enzyme in intracellular compartments, such as endosomes.

On the contrary, influenza virus replication in lung epithelial cells was shown to be dependent on NOX4-derived ROS that activates redox-sensitive intracellular pathways, including p38 and ERK1/2 MAP kinases (2). The activation of these pathways by NOX4 was proposed to promote influenza infection. While the study by Amatore *et al.* provides evidence for a virus-dependent elevation in NOX4 mRNA expression and somewhat modest elevations in ROS, the upregulation of NOX4 protein appears to not have been fully validated, given that the NOX4 commercially available antibodies are generally not recommended for use (1) and the precise antibody used by Amatore *et al.* was not described. The subcellular localization of NOX4 is a somewhat contentious issue. There is consensus that NOX4 resides in the ER forming a heterodimer with p22phox, which is critical for its activity. However, less substantiated is mitochondrial expression of NOX4 (5, 6). At this site, NOX4 is thought to act independent of p22phox, and therefore, its regulation is largely unknown. Overall, there appears to be a lack of information on the role of NOX4 in pathogen control, particularly *in vivo*. This warrants further investigation, but it is important to keep in mind that NOX4 is clearly distinct from both NOX1 and NOX2 oxidase, not only from its subcellular compartmentalization but also that NOX4 functions to generate H_2_O_2_ directly, rather than superoxide. This latter point alone is likely to substantially influence the redox biology of NOX4.

NOX1 is highly expressed in the colon, particularly in epithelial cells, and similar to phagocytic NOX2, NOX1 functions to produce superoxide. Despite its high expression and the fact that the colon is exposed to a multitude of bacteria, antimicrobial host defense response or function has not been described to date. Instead, NOX1 plays a role in cell proliferation and in redox signaling in a variety of vascular endothelial and smooth muscle cells. Finally, while there is strong evidence for endosomal localization of NOX1 protein (28), the role of NOX1 within this compartment in the context of pathogen control is completely undefined.

## Concluding Remarks

ROS biology has come a long way since the days when McCord and Fridovich discovered SOD and brought to life this exciting field (26). This perspective has focused on ROS biology that pertains to pathogen control and we know now that ROS are not only involved in these critical immune system functions but also play a role in many cellular signaling processes both physiological and pathological in nature. There is no doubt that ROS biology is a complex field, from the many chemical species that exist in nature, the multitude of enzymes and nonenzymatic systems that generate them, to the many antioxidant systems that regulate the overall levels and thus their cellular targets and functions. Although we have focused on four cellular compartments of ROS generation, that is, extracellular, phagosomal, endosomal, and mitochondrial, this is a simplistic view that was taken only to exemplify the point of compartmentalization because ROS also exist within the nucleus, ER, and cytosol. Nevertheless, the four focused compartments are important sites of ROS generation in response to invading pathogens. An emerging picture can be painted and perhaps we can suggest a unifying hypothesis: ROS production and signaling are governed by a specific invading pathogen, that is, bacteria or fungi in phagosomes and viruses in endosomes, and perhaps all converge to the powerhouse of the cell, the mitochondria, which appears to regulate both antibacterial/fungal and antiviral signaling networks (Fig. 4). The concept that the size of the invading pathogen regulates ROS compartmentalization is intriguing and perhaps speaks toward an implicit property of the innate immune system, that is, a nonspecialized, but powerful means of regulating pathogen control when the precise identity of the pathogen is not recognized.

## Road Map to Future Research Initiatives in ROS Biology

There are still major knowledge gaps in ROS biology and while the following is not an exhaustive list, these issues need to be addressed. (i) Are phagosome/endosome ROS also signaling molecules? (ii) What are the processes that facilitate ROS movement across a membrane, what are the driving forces for this to occur, and perhaps most importantly, what are the targets for this ROS in the cytosol? Are there other receptor targets in the endosome that also modulate immune function? However, if ROS biology is to progress, there needs to be a stronger push for the use of more sophisticated approaches to understand this complex biology. Studies that implicate ROS need to be more specific about what species of ROS are involved, through more specific ROS detection probes (15, 18); they must address compartmentalization of ROS, source of ROS, and target of ROS with biochemical assays and advanced imaging modalities. Armed with this type of information, we can address how these processes occur physiologically and how they contribute to the pathology associated with inflammation due to infection. Indeed, the study by To *et al.*, exemplifies how knowledge of ROS compartmentalization can be exploited for the treatment of viral infection. It demonstrates, following viral infection, the biochemical pathways leading to ROS production, the source of ROS (*i.e.*, NOX2 oxidase), the compartment of ROS production (*i.e.*, endosome), and the subcellular target of ROS (TLR7) that impact antiviral immunity. With this information, an endosome-targeted drug delivery system was developed to block endosome ROS production to virus infection. An innovative molecular targeting system was synthesized, to deliver one of the most specific NOX2 oxidase inhibitors available that was generated originally by Pagano and colleagues (*i.e.*, gp91ds-TAT) (35). The endosomally targeted system composed of gp91ds-TAT conjugated *via* a PEG-linker to cholestanol. Strikingly, targeting the biological ROS process in endosomes with cholestanol-conjugated gp91ds-TAT markedly reduced disease severity caused by influenza A virus infection. Antioxidant supplementation has been explored to alleviate oxidative stress and the burden of ROS in many disease states such as cardiovascular disease and cancer (36). Disappointingly, however, intervention trials with antioxidants have been mostly either ineffective or even harmful and to a great deal this has tainted the oxidative stress hypothesis as a culprit disease mechanisms in humans. To circumvent the concerns regarding antioxidant therapy, the more current and advanced approach is to use pharmacological agents that selectively suppress the activity of ROS forming or toxifying enzymes and whose activity or expression is increased under pathological conditions. We support this emerging paradigm but would like to advance it even further and suggest strongly that there should be a push toward spatial-specific inhibition of ROS production to ensure that physiological ROS function is unaffected while pathological ROS is suppressed.

## Abbreviations Used

ASC: apoptosis-associated speck-like protein containing a caspase recruitment domain
CGD: chronic granulomatous disease
DNA: deoxyribonucleic acid
DPI: diphenyleneiodonium chloride
DUOX2: dual oxidase 2
ER: endoplasmic reticulum
ECSIT: evolutionarily conserved signaling intermediate in toll pathways
ETC: electron transport chain
FAD: flavin adenine dinucleotide
GTP: guanosine triphosphate
H_2_O_2_: hydrogen peroxide
HOCl: hypochlorous acid
IAV: influenza A virus
IFN-α: interferon alpha
IFN-β: interferon beta
IL-1β: interleukin-1 beta
IRF-7: interferon regulatory factor 7
LPS: lipopolysaccharide
MAVS: mitochondrial antiviral signaling
MDA-5: melanoma differentiation-associated protein 5
MPO: myeloperoxidase
mtROS: mitochondrial ROS
NADPH: nicotinamide adenine dinucleotide phosphate
NF-κB: nuclear factor kappa B
NLRP3: NACHT, LRR, and PYD domains-containing protein 3
NO: nitric oxide
NOX: NADPH oxidase
ONOO^−^: peroxynitrite
PKC: protein kinase C
RAC: ras-related C3 botulinum toxin substrate
RET: reversal of electron transport
RIG-I: retinoic acid inducible gene I
RNA: ribonucleic acid
ROS: reactive oxygen species
SOD: superoxide dismutase
TLR: toll-like receptor
TRAF: tumor necrosis factor receptor-associated factor

## References

[1] Altenhofer S, Radermacher KA, Kleikers PW, Wingler K, and Schmidt HH Evolution of NADPH oxidase inhibitors: selectivity and mechanisms for target engagement. Antioxid Redox Signal 23: 406–427, 2015 24383718 10.1089/ars.2013.5814 PMC4543484

[2] Amatore D, Sgarbanti R, Aquilano K, Baldelli S, Limongi D, Civitelli L, Nencioni L, Garaci E, Ciriolo MR, and Palamara AT Influenza virus replication in lung epithelial cells depends on redox-sensitive pathways activated by NOX4-derived ROS. Cell Microbiol 17: 131–145, 2015 25154738 10.1111/cmi.12343 PMC4311438

[3] Babior BM. NADPH oxidase: an update. Blood 93: 1464–1476, 1999 10029572

[4] Baldridge CW and Gerard RW The extra respiration of phagocytosis. Am J Physiol 103: 235, 1932

[5] Bedard K and Krause KH The NOX family of ROS-generating NADPH oxidases: physiology and pathophysiology. Physiol Rev 87: 245–313, 2007 17237347 10.1152/physrev.00044.2005

[6] Block K, Gorin Y, and Abboud HE Subcellular localization of Nox4 and regulation in diabetes. Proc Natl Acad Sci U S A 106: 14385–14390, 2009 19706525 10.1073/pnas.0906805106 PMC2732863

[7] Brandes RP, Weissmann N, and Schroder K Nox family NADPH oxidases: molecular mechanisms of activation. Free Radic Biol Med 76: 208–226, 2014 25157786 10.1016/j.freeradbiomed.2014.07.046

[8] Buskiewicz IA, Montgomery T, Yasewicz EC, Huber SA, Murphy MP, Hartley RC, Kelly R, Crow MK, Perl A, Budd RC, and Koening A Reactive oxygen species induce virus-independent MAVS oligomerization in systemic lupus erythematosus. Sci Signal 9: ra115, 2016 27899525 10.1126/scisignal.aaf1933 PMC5321043

[9] Dostert C, Petrilli V, Van Bruggen R, Steele C, Mossman BT, and Tschopp J Innate immune activation through Nalp3 inflammasome sensing of asbestos and silica. Science 320: 674–677, 2008 18403674 10.1126/science.1156995 PMC2396588

[10] Drummond GR, Selemidis S, Griendling KK, and Sobey CG Combating oxidative stress in vascular disease: NADPH oxidases as therapeutic targets. Nat Rev Drug Discov 10: 453–471, 2011 21629295 10.1038/nrd3403 PMC3361719

[11] Geiszt M and Leto TL The Nox family of NAD(P)H oxidases: host defense and beyond. J Biol Chem 279: 51715–51718, 2004 15364933 10.1074/jbc.R400024200

[12] Geiszt M, Witta J, Baffi J, Lekstrom K, and Leto TL Dual oxidases represent novel hydrogen peroxide sources supporting mucosal surface host defense. FASEB J 11: 1502–1504, 2003 10.1096/fj.02-1104fje 12824283

[13] Geng J, Sun X, Wang P, Zhang S, Wang X, Wu H, Hong L, Xie C, Li X, Zhao H, Liu Q, Jiang M, Chen Q, Zhang J, Li Y, Song S, Wang HR, Zhou R, Johnson RL, Chien KY, Lin SC, Han J, Avruch J, Chen L, and Zhou D Kinases Mst1 and Mst2 positively regulate phagocytic induction of reactive oxygen species and bactericidal activity. Nat Immunol 16: 1142–1152, 2015 26414765 10.1038/ni.3268 PMC4618176

[14] Grasberger H, El-Zaatari M, Dang DT, and Merchant JL Dual oxidases control release of hydrogen peroxide by the gastric epithelium to prevent Helicobacter felis infection and inflammation in mice. Gastroenterology 145: 1045–1054, 2013 23860501 10.1053/j.gastro.2013.07.011 PMC3805753

[15] Griendling KK, Touyz RM, Zweier JL, Dikalov S, Chilian W, Chen YR, Harrison DG, Bhatnagar A; American Heart Association Council on Basic Cardiovascular Sciences Measurement of reactive oxygen species, reactive nitrogen species, and redox-dependent signaling in the cardiovascular system: a scientific statement from the American Heart Association. Circ Res 119: e39–e75, 2016 27418630 10.1161/RES.0000000000000110 PMC5446086

[16] Imai Y, Kuba K, Neely GG, Yaghubian-Malhami R, Perkmann T, van Loo G, Ermolaeva M, Veldhuizen R, Leung YH, Wang H, Liu H, Sun Y, Pasparakis M, Kopf M, Mech C, Bavari S, Peiris JS, Slutsky AS, Akira S, Hultqvist M, Holmdahl R, Nicholls J, Jiang C, Binder CJ, and Penninger JM Identification of oxidative stress and Toll-like receptor 4 signaling as a key pathway of acute lung injury. Cell 133: 235–249, 2008 18423196 10.1016/j.cell.2008.02.043 PMC7112336

[17] Jeon YJ and Kim HJ Duox2-induced innate immune responses in the respiratory epithelium and intranasal delivery of Duox2 DNA using polymer that mediates immunization. Appl Microbiol Biotechnol 102: 4339–4343, 2018 29600494 10.1007/s00253-018-8956-y

[18] Kalyanaraman B, Darley-Usmar V, Davies KJ, Dennery PA, Forman HJ, Grisham MB, Mann GE, Moore K, Roberts LJ, 2nd, and Ischiropoulos H Measuring reactive oxygen and nitrogen species with fluorescent probes: challenges and limitations. Free Radic Biol Med 52: 1–6, 2012 22027063 10.1016/j.freeradbiomed.2011.09.030 PMC3911769

[19] Kim HJ, Kim CH, Kim MJ, Ryu JH, Seong SY, Kim S, Lim SJ, Holtzman MJ, and Yoon JH The induction of pattern-recognition receptor expression against influenza A virus through Duox2-derived reactive oxygen species in nasal mucosa. Am J Respir Cell Mol Biol 53: 525–535, 2015 25751630 10.1165/rcmb.2014-0334OC PMC5455469

[20] Kim HJ, Kim CH, Ryu JH, Kim MJ, Park CY, Lee JM, Holtzman MJ, and Yoon JH Reactive oxygen species induce antiviral innate immune response through IFN-λ regulation in human nasal epithelial cells. Am J Respir Cell Mol Biol 49: 855–865, 2013 23786562 10.1165/rcmb.2013-0003OC PMC5455605

[21] Lamb FS, Moreland JG, and Miller FJ Jr Electrophysiology of reactive oxygen production in signaling endosomes. Antioxid Redox Signal 11: 1335–1347, 2009 19207039 10.1089/ars.2008.2448 PMC2872256

[22] Lambeth JD. NOX enzymes and the biology of reactive oxygen. Nat Rev Immunol 4: 181–189, 2004 15039755 10.1038/nri1312

[23] Lejal N, Truchet S, Bechor E, Bouguyon E, Khedkar V, Bertho N, Vidic J, Adenot P, Solier S, Pick E, and Slama-Schwok A Turning off NADPH oxidase-2 by impeding p67phox activation in infected mouse macrophages reduced viral entry and inflammation. Biochim Biophys Acta 1862: 1263–1275, 2018 10.1016/j.bbagen.2018.03.004 29524539

[24] Li Q, Harraz MM, Zhou W, Zhang LN, Ding W, Zhang Y, Eggleston T, Yeaman C, Banfi B, and Engelhardt JF Nox2 and Rac1 regulate H2O2-dependent recruitment of TRAF6 to endosomal interleukin-1 receptor complexes. Mol Cell Biol 26: 140–154, 2006 16354686 10.1128/MCB.26.1.140-154.2006 PMC1317618

[25] Marriott HM, Jackson LE, Wilkinson TS, Simpson AJ, Mitchell TJ, Buttle DJ, Cross SS, Ince PG, Hellewell PG, Whyte MK, and Dockrell DH Reactive oxygen species regulate neutrophil recruitment and survival in pneumococcal pneumonia. Am J Respir Crit Care Med 177: 887–895, 2008 18202350 10.1164/rccm.200707-990OC PMC2643216

[26] McCord JM and Fridovich I Superoxide dismutase. An enzymic function for erythrocuprein (hemocuprein). J Biol Chem 244: 6049–6055, 1969 5389100

[27] Meissner F, Molawi K, and Zychlinsky A Superoxide dismutase 1 regulates caspase-1 and endotoxic shock. Nat Immunol 9: 866–872, 2008 18604212 10.1038/ni.1633

[28] Meissner F, Seger RA, Moshous D, Fischer A, Reichenbach J, and Zychlinsky A Inflammasome activation in NADPH oxidase defective mononuclear phagocytes from patients with chronic granulomatous disease. Blood 116: 1570–1573, 2010 20495074 10.1182/blood-2010-01-264218 PMC2938844

[29] Miller FJ Jr., Filali M, Huss GJ, Stanic B, Chamseddine A, Barna TJ, and Lamb FS. Cytokine activation of nuclear factor kappa B in vascular smooth muscle cells requires signaling endosomes containing Nox1 and ClC-3. Circ Res 101: 663–671, 2007 17673675 10.1161/CIRCRESAHA.107.151076

[30] Mills EL, Kelly B, Logan A, Costa AS, Varma M, Bryant CE, Tourlomousis P, Dabritz JH, Gottlieb E, Latorre I, Corr SC, McManus G, Ryan D, Jacobs HT, Szibor M, Xavier RJ, Braun T, Frezza C, Murphy MP, and O'Neill LA Succinate dehydrogenase supports metabolic repurposing of mitochondria to drive inflammatory macrophages. Cell 167: 457–470.e413, 2016 27667687 10.1016/j.cell.2016.08.064 PMC5863951

[31] Mills EL, Kelly B, and O'Neill LAJ Mitochondria are the powerhouses of immunity. Nat Immunol 18: 488–498, 2017 28418387 10.1038/ni.3704

[32] Mumbengegwi DR, Li Q, Li C, Bear CE, and Engelhardt JF Evidence for a superoxide permeability pathway in endosomal membranes. Mol Cell Biol 28: 3700–3712, 2008 18378695 10.1128/MCB.02038-07 PMC2423302

[33] Nauseef WM. How human neutrophils kill and degrade microbes: an integrated view. Immunol Rev 219: 88–102, 2007 17850484 10.1111/j.1600-065X.2007.00550.x

[34] Reeves EP, Lu H, Jacobs HL, Messina CG, Bolsover S, Gabella G, Potma EO, Warley A, Roes J, and Segal AW Killing activity of neutrophils is mediated through activation of proteases by K+ flux. Nature 416: 291–297, 2002 11907569 10.1038/416291a

[35] Rey FE, Cifuentes ME, Kiarash A, Quinn MT, and Pagano PJ Novel competitive inhibitor of NAD(P)H oxidase assembly attenuates vascular O(2)(−) and systolic blood pressure in mice. Circ Res 89: 408–414, 2001 11532901 10.1161/hh1701.096037

[36] Schmidt HH, Stocker R, Vollbracht C, Paulsen G, Riley D, Daiber A, and Cuadrado A Antioxidants in translational medicine. Antioxid Redox Signal 23: 1130–1143, 2015 26154592 10.1089/ars.2015.6393 PMC4657516

[37] Segal AW. How neutrophils kill microbes. Annu Rev Immunol 23: 197–223, 2005 15771570 10.1146/annurev.immunol.23.021704.115653 PMC2092448

[38] Selemidis S, Seow HJ, Broughton BR, Vinh A, Bozinovski S, Sobey CG, Drummond GR, and Vlahos R Nox1 oxidase suppresses influenza a virus-induced lung inflammation and oxidative stress. PLoS One 8: 4, 2013 10.1371/journal.pone.0060792 PMC3620107 23577160

[39] Selemidis S, Sobey CG, Wingler K, Schmidt HH, and Drummond GR NADPH oxidases in the vasculature: molecular features, roles in disease and pharmacological inhibition. Pharmacol Ther 120: 254–291, 2008 18804121 10.1016/j.pharmthera.2008.08.005

[40] Strengert M, Jennings R, Davanture S, Hayes P, Gabriel G, and Knaus UG Mucosal reactive oxygen species are required for antiviral response: role of Duox in influenza a virus infection. Antioxid Redox Signal 20: 2695–2709, 2014 24128054 10.1089/ars.2013.5353

[41] Snelgrove RJ, Edwards L, Rae AJ, and Hussell T An absence of reactive oxygen species improves the resolution of lung influenza infection. Eur J Immunol 36: 1364–1373, 2006 16703568 10.1002/eji.200635977

[42] Szabo C, Ischiropoulos H, and Radi R Peroxynitrite: biochemistry, pathophysiology and development of therapeutics. Nat Rev Drug Discov 6: 662–680, 2007 17667957 10.1038/nrd2222

[43] To EE, Vlahos R, Luong R, Halls ML, Reading PC, King PT, Chan C, Drummond GR, Sobey CG, Broughton BRS, Starkey MR, van der Sluis R, Lewin SR, Bozinovski S, O'Neill LAJ, Quach T, Porter CJH, Brooks DA, O'Leary JJ, and Selemidis S Endosomal NOX2 oxidase exacerbates virus pathogenicity and is a target for antiviral therapy. Nat Commun 8: 69, 2017 28701733 10.1038/s41467-017-00057-x PMC5507984

[44] van Bruggen R, Koker MY, Jansen M, van Houdt M, Roos D, Kuijpers TW, and van den Berg TK Human NLRP3 inflammasome activation is Nox1–Nox4 independent. Blood 115: 5398–5400, 2010 20407038 10.1182/blood-2009-10-250803

[45] van der Vliet A, Danyal K, and Heppner DE Dual oxidase: a novel therapeutic target in allergic disease. Br J Pharmacol 175: 1401–1418, 2018 29405261 10.1111/bph.14158 PMC5900994

[46] Vlahos R, Stambas J, Bozinovski S, Broughton BR, Drummond GR, and Selemidis S Inhibition of Nox2 oxidase activity ameliorates influenza A virus-induced lung inflammation. PLoS Pathog 7: e1001271, 2011 21304882 10.1371/journal.ppat.1001271 PMC3033375

[47] Vlahos R, Stambas J, and Selemidis S Suppressing production of reactive oxygen species (ROS) for influenza A virus therapy. Trends Pharmacol Sci 33: 3–8, 2012 21962460 10.1016/j.tips.2011.09.001

[48] Warnatsch A, Tsourouktsoglou TD, Branzk N, Wang Q, Reincke S, Herbst S, Gutierrez M, and Papayannopoulos V Reactive oxygen species localization programs inflammation to clear microbes of different size. Immunity 46: 421–432, 2017 28314592 10.1016/j.immuni.2017.02.013 PMC5965455

[49] West AP, Brodsky IE, Rahner C, Woo DK, Erdjument-Bromage H, Tempst P, Walsh MC, Choi Y, Shadel GS, and Ghosh S TLR signalling augments macrophage bactericidal activity through mitochondrial ROS. Nature 472: 476–480, 2011 21525932 10.1038/nature09973 PMC3460538

[50] Winterbourn CC. Reconciling the chemistry and biology of reactive oxygen species. Nat Chem Biol 4: 278–286, 2008 18421291 10.1038/nchembio.85

[51] Winterbourn CC, Kettle AJ, and Hampton MB Reactive oxygen species and neutrophil function. Annu Rev Biochem 85: 765–792, 2016 27050287 10.1146/annurev-biochem-060815-014442

[52] Zhang Z, Ohto U, Shibata T, Krayukhina E, Taoka M, Yamauchi Y, Tanji H, Isobe T, Uchiyama S, Miyake K, and Shimizu T Structural analysis reveals that Toll-like receptor 7 is a dual receptor for guanosine and single-stranded RNA. Immunity 45: 737–748, 2016 27742543 10.1016/j.immuni.2016.09.011

[53] Zhou R, Yazdi AS, Menu P, and Tschopp J A role for mitochondria in NLRP3 inflammasome activation. Nature 469: 221–225, 2011 21124315 10.1038/nature09663

